# Structural and phylogenetic implications of the complete mitochondrial genome of *Ledra auditura*

**DOI:** 10.1038/s41598-019-52337-9

**Published:** 2019-10-31

**Authors:** Jia-Jia Wang, De-Fang Li, Hu Li, Mao-Fa Yang, Ren-Huai Dai

**Affiliations:** 1 The Provincial Key Laboratory for Agricultural Pest Management Mountainous Region, Institute of Entomology, Guizhou University, Guiyang, Guizhou 550025 P.R. China; 2 Shaanxi Key Laboratory of Bioresources, Shaanxi University of Technology, Hanzhong, Shaanxi 723000 P.R. China

**Keywords:** Sequence annotation, Entomology

## Abstract

We sequenced and annotated the first complete mitochondrial genome (mitogenome) of *Ledra auditura* (Hemiptera: Cicadellidae: Ledrinae) and reconstructed phylogenetic relationships among 47 species (including 2 outgroup species) on the basis of 3 datasets using maximum likelihood (ML) and Bayesian inference (BI) analyses. The complete *L. auditura* mitogenome (length, 16,094 bp) comprises 37 genes [13 protein-coding genes (PCGs), 22 tRNAs, and 2 rRNAs], 1 control region, and 2 long non-coding regions. The first long non-coding region (length, 211 bp) is located between *tRNA-I* and *tRNA-Q* and the second region (length, 994 bp) between *tRNA-S2* and *ND1*. All PCGs show ATN (Met/Ile) as their start codon and TAR as their stop codon. Except *tRNA-S1* (AGN), which lacks the dihydrouridine arm, all tRNAs can fold into the typical cloverleaf secondary structure. The complete *L. auditura* mitogenome shows a base composition bias of 76.3% A + T (A = 29.9%, T = 46.4%, G = 13.3%, and C = 10.5%), negative AT skew of −0.22, and positive GC skew of 0.12. In ML and BI analyses, *L. auditura* was clustered with *Evacanthus heimianus* (Hemiptera: Cicadellidae: Evacanthinae) with strong branch support.

## Introduction

Ledrinae is a relatively small subfamily within the large and diverse leafhopper family Cicadellidae, which comprises approximately 300 described species of 38 genera divided into 5 tribes. These species are extensively distributed across Australia, Africa, and Southeast Asia^1^ and primarily inhabit trees and shrubs, except for the grass-feeding Xerophloeini^2^. Moreover, no Ledrinae species appear to be major vectors of plant diseases^3^. A few members of Ledrinae show conspicuous ear-like projections on the pronotum and are referred to as eared leafhoppers^4^. Interest in Ledrinae is often centered on this unique morphology along with its possible implications in the evolutionary history of leafhoppers^5,6^.

Continuous improvements and advancements in molecular biology techniques have facilitated wide use of high-throughput sequencing for mitochondrial genome (mitogenome) data collection. Complete mitogenomes have been effectively used to understand the evolutionary relationships among insects^7–9^. Since 2016, there has been a drastic increase in the availability of Cicadellidae mitogenome data; however, despite their great diversity, only 40 complete or near-complete Cicadellidae mitogenomes have been reported to date^10–27^. Therefore, new mitogenomic data will provide support for determining the phylogenetic relationships and evolution of Cicadellidae in the future. Here, using first- and second-generation sequencing, we sequenced the complete mitogenome of *Ledra auditura* to confirm its phylogenetic relationships and taxonomic status as well as to better understand its mitogenome structure. *L. auditura* is the first Ledrinae species whose phylogenetic relationships with 40 other leafhoppers and 5 treehoppers have been assessed using maximum likelihood (ML) and Bayesian inference (BI) analyses based on mitogenomes, thereby providing a basis for further molecular research on the related taxa.

## Results and Discussion

### Genome organization and base composition

**Figure 1:**
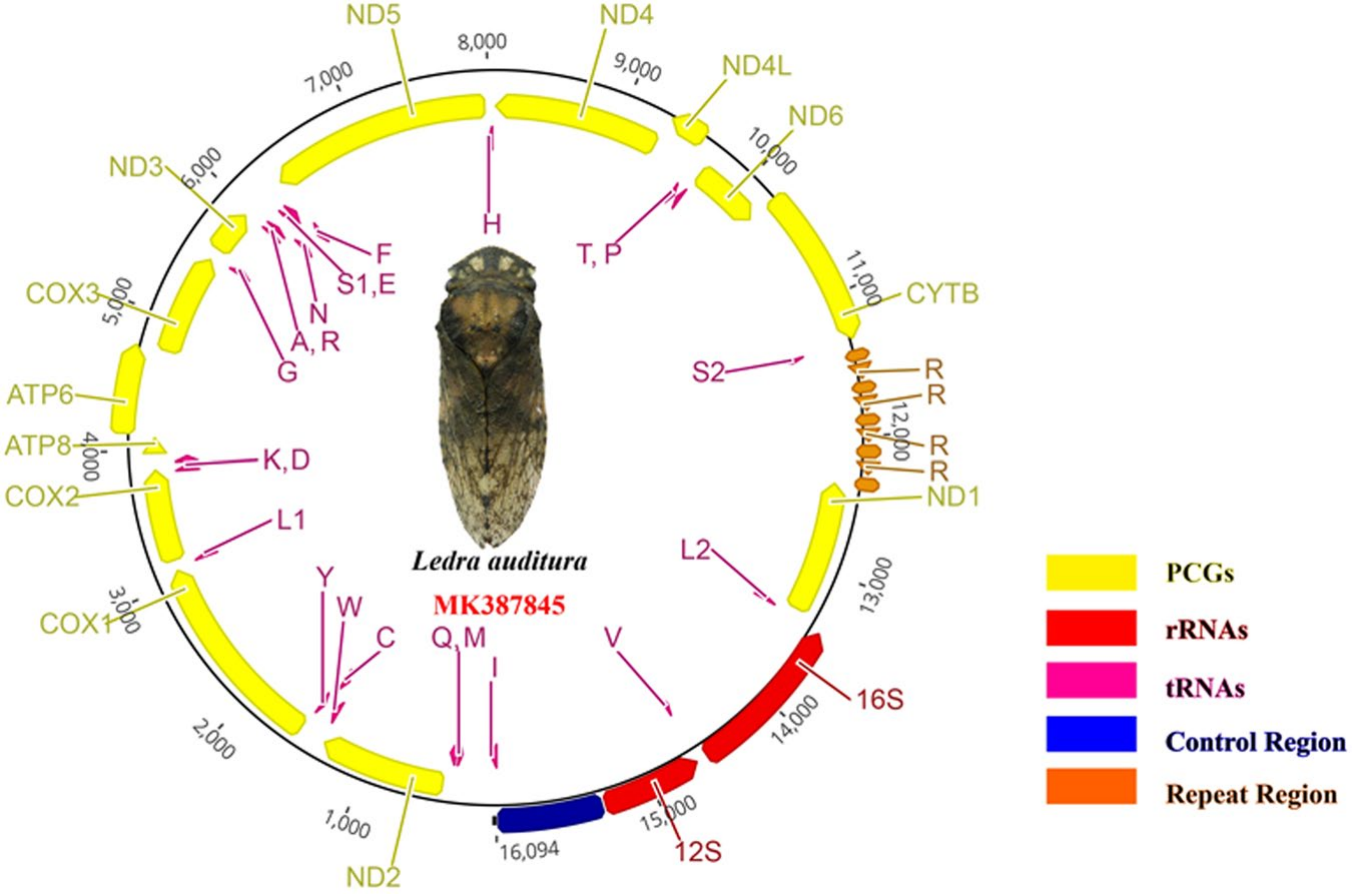
Circular map of the *Ledra auditura* mitogenome.

The complete *L. auditura* mitogenome (GenBank No., MK387845) is 16,094-bp long, which is comparable to the sizes of previously documented mitogenomes of Cicadellidae species, ranging from 15,131 of *Trocnadella arisana* to 16,811 bp of *Parocerus laurifoliae*^25^. The gene order and arrangement of the *L. auditura* mitogenome are identical to those of other commonly sequenced Hemiptera species^8–19^. A total of 22 genes (9 PCGs and 13 tRNAs) are encoded on the majority strand (J-strand) and 15 (4 PCGs, 9 tRNAs, and 2 rRNAs) on the minority strand (N-strand) (Fig. 1, Table 1). However, there are 2 long non-coding regions in *L. auditura* in addition to the control region; the first region (length, 211 bp) is located between *tRNA-I* and *tRNA-Q* and the second (length, 994 bp) between *tRNA-S2* and *ND1*. The nucleotide composition of the complete mitogenome is as follows: A = 29.9%, T = 46.4%, G = 13.3%, and C = 10.5%. The complete *L. auditura* mitogenome shows a base composition bias of 76.3% A + T, a negative AT skew of −0.22, and a positive GC skew of 0.12 (Table 2).

**Table 1:** Organization of the *Ledra auditura* mitogenome.

Name	Direction	Location	Size (bp)	Anti/Start/Stop codon	Intergenic nucleotides
*tRNA-I*	F	1–65	65	30–32 GAT	
*tRNA-Q*	R	277–342	66	312–310 TTG	211
*tRNA-M*	F	344–410	67	376–378 CAT	1
*ND2*	F	411–1,385	975	ATT/TAA	0
*tRNA-W*	F	1,386–1,450	65	1,418–1,420 TCG	0
*tRNA-C*	R	1,443–1,505	63	1,477–1,475 GCA	−8
*tRNA-Y*	R	1,505–1,570	66	1,541–1,539 GTA	−1
*COX1*	F	1,571–3,106	1,536	ATG/TAG	0
*tRNA-L1*	F	3,107–3,174	68	3,139–3,141 TAA	0
*COX2*	F	3,175–3,882	708	ATT/TAA	0
*tRNA-K*	F	3,848–3,918	71	3,878–3,880 CTT	−35
*tRNA-D*	F	3,923–3,985	63	3,953–3,955 GTC	4
*ATP8*	F	3,982–4,131	150	ATA/TAA	−4
*ATP6*	F	4,135–4,762	628	ATT/TAG	3
*COX3*	F	4,756–5,553	798	ATT/TAA	−7
*tRNA-G*	F	5,554–5,616	63	5,586–5,588 TCC	0
*ND3*	F	5,614–5,970	357	ATA/TAG	−3
*tRNA-A*	F	5,969–6,029	61	5,998–6,000 TGC	−2
*tRNA-R*	F	6,031–6,091	61	6,058–6,060 TCG	1
*tRNA-N*	F	6,091–6,152	62	6,119–6,121 GTT	−1
*tRNA-S1*	F	6,153–6,213	61	6,174–6,176 GCT	0
*tRNA-E*	F	6,214–6,275	62	6,244–6,246 TTC	0
*tRNA-F*	R	6,272–6,333	62	6,303–6,301 GAA	−4
*ND5*	R	6,317–7,999	1,683	ATC/TAA	−17
*tRNA-H*	R	7,997–8,058	62	8,027–8,025 GTG	−3
*ND4*	R	8,059–9,324	1,266	ATA/TAA	0
*ND4L*	R	9,324–9,599	276	ATG/TAA	−1
*tRNA-T*	F	9,600–9,661	62	9,630–9,632 TGT	0
*tRNA-P*	R	9,662–9,724	63	9,694–9,692 TGG	0
*ND6*	F	9,703–10,236	504	ATT/TAA	−22
*Cytb*	F	10,217–11,350	1,134	ATG/TAG	−20
*tRNA-S2*	F	11,349–11,411	63	11,379–11,381 TGA	−2
Repeat region		11,410–12,402	993		−2
*ND1*	R	12,359–13,363	1,005	ATA/TAA	−44
*tRNA-L2*	R	13,361–13,427	67	13,396–13,394 TAG	−3
*16S*	R	13,428–14,587	1,160		0
*tRNA-V*	R	14,588–14,652	65	14,619–14,617 TAC	0
*12S*	R	14,653–15,373	721		0
CR		15,374–16,094	721		0

NOTE: Intergenic nucleotides indicate gap (positive value) or overlapping nucleotides (negative value) between 2 adjacent genes. CR: Control region.

**Table 2:** Nucleotide composition of the *Ledra auditura* mitogenome.

Feature	A%	T%	G%	C%	A + T	AT skew	GC skew
Whole mitogenome	29.9	46.4	13.3	10.5	76.3	−0.22	0.12
PCGs	29.3	45.3	13.5	11.8	74.6	−0.21	0.07
tRNAs	37.6	39.7	12.6	10.1	77.3	−0.03	0.11
rRNAs	45.5	33.4	12.0	9.1	78.9	0.15	0.14
Control region	44.8	46.3	6.8	5.1	91.1	−0.02	0.14

PCGs: protein-coding genes.

### PCGs and codon usage

The total length of the 13 PCGs is 11,064 bp, and these encode 3,688 amino acids, accounting for 68.7% of the complete *L. auditura* mitogenome. All PCGs are initially encoded by ATN (Met/Ile). The start codon of 4 genes (*ND2*, *COX2*, *COX3*, and *ND6*) is ATT, that of 4 other genes (*ATP8*, *ND3*, *ND4*, and *ND1*) is ATA, that of 1 gene (*ND5*) is ATC, and that of the remaining 4 genes (*COX1*, *ATP6*, *ND4L*, and *Cytb*) is ATG. The stop codon of 9 PCGs is the typical TAA and that of 4 (*COX1*, *ATP6*, *ND3*, and *Cytb*) PCGs is TAG (Table 3).

**Table 3:** Primers used for mitogenome analysis.

NO.	Primer sequence (5′–3′)	Annealing temperature	Amplified region
1	F: GGTCAACAAATCATAAAGATATTGG	50 °C	*COX1* (1,611–2,270 bp)
	R: TAAACTTCAGGGTGACCAAAAAATCA	50 °C	
2	F: ACGTTTCTATCGTCTTTATACT	48 °C	*tRNA-S2–ND1* (11,202–12,501 bp)
	R: TACCAATAACATTGAACATAA	48 °C	
3	F: AAAGTAAGTAATAACCGCCAAAT	48 °C	*12S–tRNA-I* (15,019–23 bp)
	R: CTTTATTCAGGCACTTTACTTTAT	48 °C	

F: forward; R: reverse.

**Figure 2:**
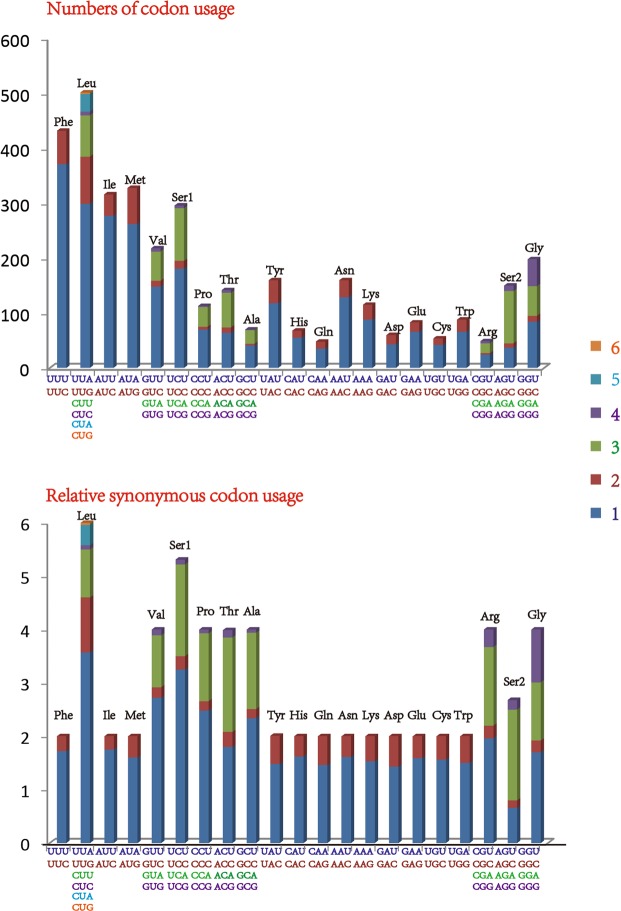
Relative synonymous codon usage and number of codon usage in the *Ledra auditura* mitogenome Codon families are indicated on the X-axis.

The base composition of the 13 PCGs is 74.6% A + T (A = 29.3%, T = 45.3%, G = 13.5%, and C = 11.8%), with a negative AT skew (−0.21) and weakly positive GC skew (0.07). The relative synonymous codon usage and codon usage of the 13 PCGs of the *L. auditura* mitogenome are presented in Fig. 2 (except the stop codons TAA and TAG). Within each codon, the third codon position terminating with A/T is more frequent than that with G/C, thereby resulting in the highest A + T content at the third codon position. The 4 most frequently used codons are Phe (TTT), Leu (TTA), Ile (ATT), and Met (ATA). In addition, codon usage exhibits a high A + T bias that plays a key role in the A + T bias of the entire mitogenome. The codon usage pattern of *L. auditura* is highly consistent with that of previously reported Cicadellidae species^8–17^.

### tRNAs and rRNAs

**Figure 3:**
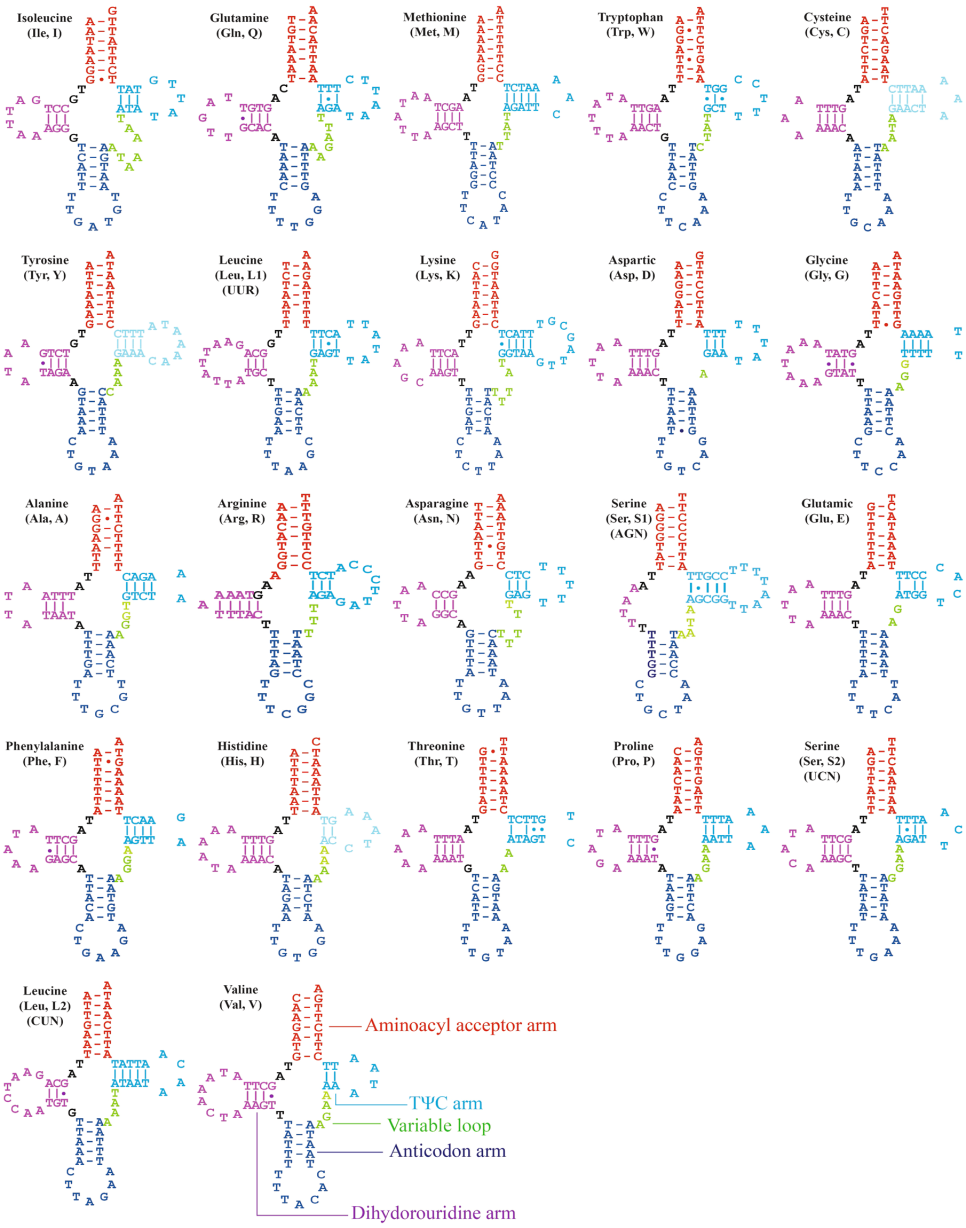
Predicted secondary structure of the 22 tRNAs in the *Ledra auditura* mitogenome; dashes (–) indicate Watson–Crick base pairing.

The *L. auditura* mitogenome comprises the 22 typical tRNAs, with lengths ranging from 61 (Ala, Arg, and Ser1) to 71 (Lys) bp (Table 1). The total length of the 22 tRNAs is 1,408 bp, with 77.3% A + T content. All tRNAs can fold into the typical cloverleaf secondary structure except *tRNA-S1*, which lacks the dihydrouridine arm, as documented for other Hemiptera species^9,28,29^. The secondary structure of the 22 tRNAs is presented in Fig. 3.

**Figure 4:**
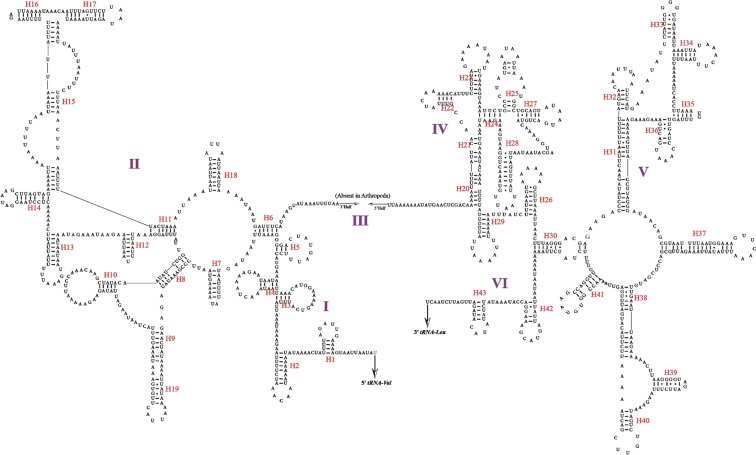
Predicted secondary structure of *16S* rRNA in the *Ledra auditura* mitogenome; dashes (–) indicate Watson–Crick base pairing.

**Figure 5:**
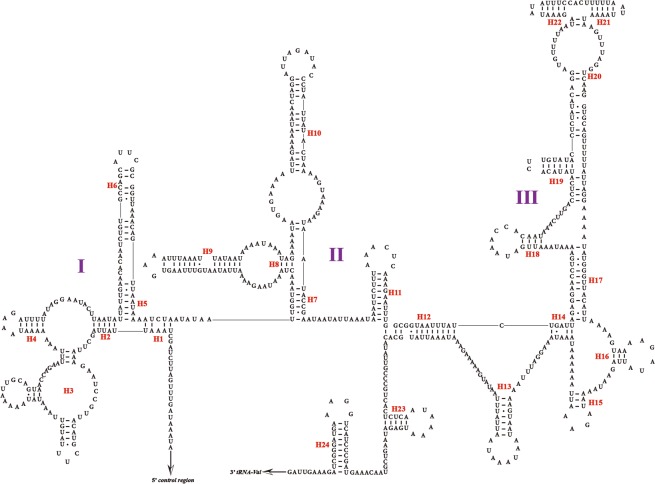
Predicted secondary structure of *12S* rRNA in the *Ledra auditura* mitogenome; dashes (–) indicate Watson–Crick base pairing.

The *16S* and *12S* rRNA genes in the Cicadellidae mitogenome are highly conserved in terms of their length and secondary structures^22–25^. In the *L. auditura* mitogenome, the *16S* rRNA is located between *tRNA-L2* and *tRNA-V* and is 1,160-bp long. The *12S* rRNA gene, as identified based on the alignments with *Evacanthus heimianus* and *Idioscopus clypealis*^13^, is located between *tRNA-V* and the control region and is 721-bp long. In the present study, the hypothetical secondary structures of 2 rRNA genes were drawn using RNA Structure version 5.2^30^, predicted against the known rRNA secondary structures^25,31,32^. The secondary structure of *16S* rRNA in the *L. auditura* mitogenome comprises 5 domains (domains I, II, IV, V, and VI; domain III is absent, as in other insects) and 43 helices (Fig. 4) and that of *12S* rRNA comprises 3 domains (domains I, II and III) and 24 helices (Fig. 5).

### Non-coding regions

**Figure 6:**
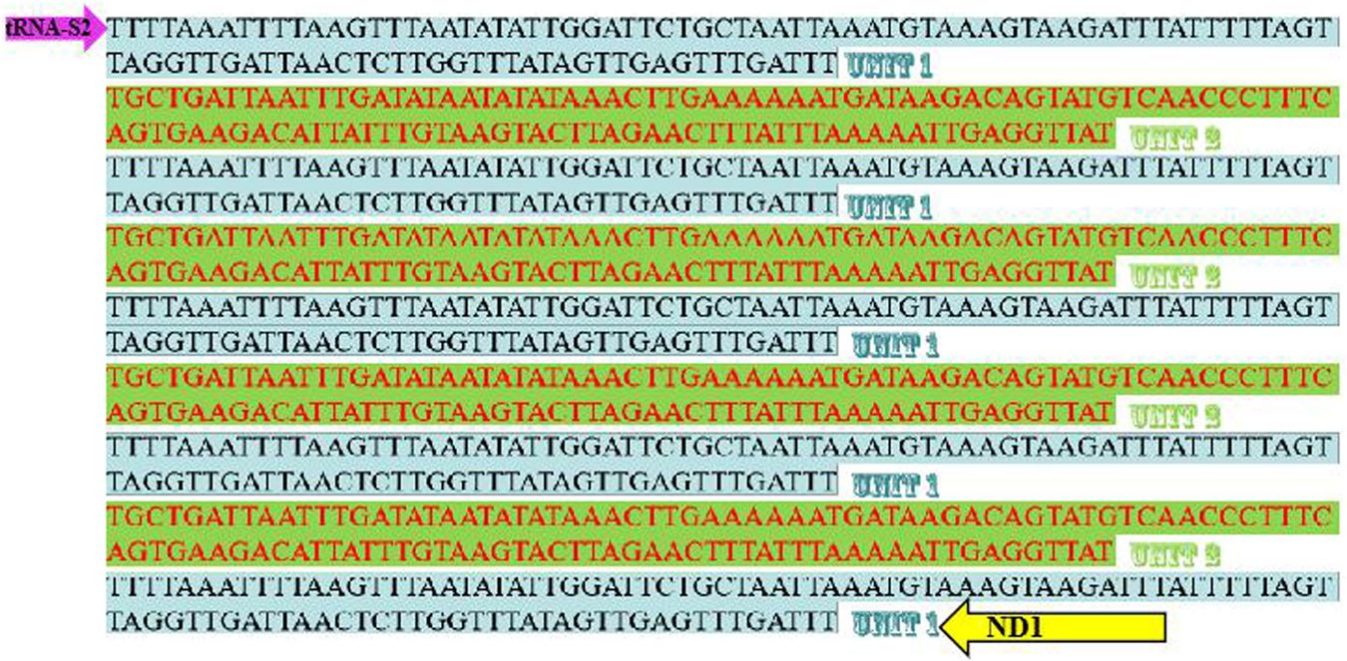
Structure of repeat regions in the *Ledra auditura* mitogenome.

Although large intergenic regions have been identified in some species, the mitogenomes of most insects are compact^33^. Usually the long non-coding region is located between *12S rRNA* and *tRNA-I*, which is the control region. In the present study, 3 long non-coding regions (>50 bp) were detected in the *L. auditura* mitogenome. The first non-coding region (length, 211 bp) is located between *tRNA-I* and *tRNA-Q*. The second non-coding region (length, 993 bp) is a repeat region located between *tRNA-S2* and *ND1*. It comprises 2 tandem repeats (Figs 1 and 6): the first repeat sequence is 105-bp long and is repeated 5 times, and the second is 117-bp long and repeated 4 times (Fig. 6). Finally, the third non-coding region, commonly referred to as the control region, is located between *12S rRNA* and *tRNA-I*; it is 721-bp long, which is comparable to that reported in other sequenced leafhoppers, ranging from399 bp of *N. cincticeps* to 2477 bp of *Parocerus laurifoliae*. The region shows 91.1% A + T content, and it is the most variable region in the whole mitogenome, with a relatively low pairwise identity. The control region is usually much longer in species with repetitive sequences than in those without repeats. However, there was no association among each repeat unit, the regularity of the occurrence of repetitive sequences, and the significance in the control area, suggesting the need for further research using different methods to resolve this pattern in the future.

### Phylogenetic relationships

**Figure 7:**
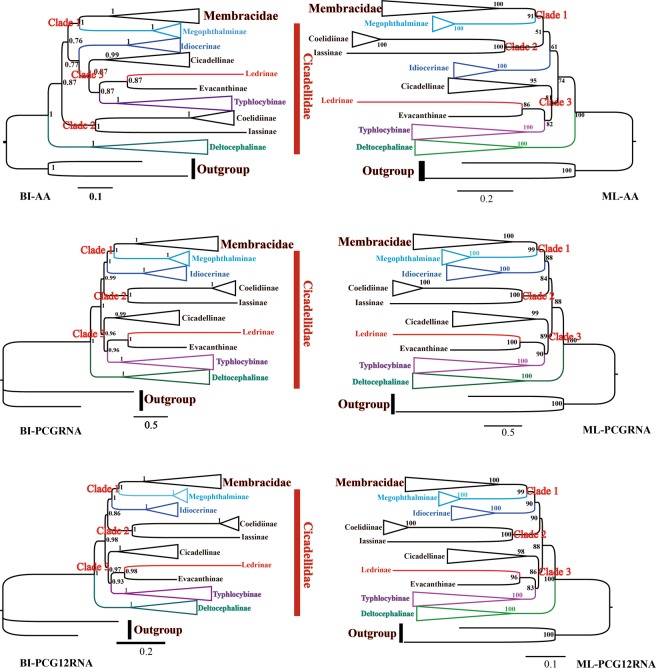
Phylogenetic trees of Cicadellidae inferred using maximum likelihood (ML) and MrBayes (BI) analyses based on protein-coding genes and rRNA genes.

**Figure 8:**
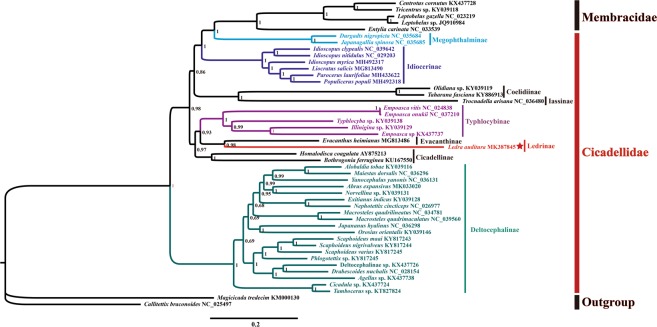
Phylogenetic trees of *Ledra auditura* constructed based on the first and second codons of the 13 PCGs and 2 rRNAs using the GTR + I + G model determined by MrBayes.

ML and BI analyses were used to reconstruct the phylogenetic relationships among 47 species (including 2 outgroup species) based on the following 3 datasets: (1) amino acid sequences of 13 PCGs (3,366 amino acids); (2) nucleotide sequences of 13 PCGs and 2 rRNAs (11,918 bp); (3) the first and second codons of 13 PCGs and complete sequences of 2 rRNAs (8,552 bp). A total of 6 phylogenetic trees (BI-AA, BI-PCGRNA, BI-PCG12RNA, ML-AA, ML-PCGRNA, and ML-PCG12RNA) reconstructed using ML and BI analyses on the 3 datasets are shown in Figs 7, 8 and S1–S5. Previous molecular phylogenetic analyses have suggested that Delocephalinae leafhoppers constitute 1 clade, which has been recovered as the sister group to the other members of Cicadellidae^22–27^. In the present study, the relationships among the 3 clades was consistent with high support in all the trees [clade 1: Membracidae + Megophthalminae; clade 2: Coelidiinae + Iassinae; clade 3: Cicadellinae + (Typhlocybinae + {Evacanthinae + Ledrinae})]; this result is consistent with previously reported phylogenies using partial gene sequences and morphological features^34–37^, suggesting that Cicadellidae is paraphyletic with treehoppers, but Cicadellinae subfamilies, including Deltocephalinae, Megophthalminae, Idiocerinae, Typhlocybinae, Cicadellinae, and Coelidiinae are monophyletic, with strong branch support. Within Cicadellidae, the inferred relationship (Iassinae + Coelidiinae) + [Deltophalinae + (Megophthalminae + Idiocerinae)] + [Cicadellinae + (Typhlocybinae + {Evacanthinae + Ledrinae})] was supported with high moderated branch support in 4 phylogenetic trees (BI-PCGRNA, BI-PCG12RNA, ML-PCGRNA and ML-PCG12RNA) (Figs 7, 8, and S3–S5), but Idiocerinae was recovered as the sister clade to Cicadellinae + (Typhlocybinae + (Evacanthinae + Ledrinae)) in BI-AA and (Membracidae + Megophthalminae) + (Coelidiinae + Iassinae) in ML-AA, with low branch support (Figs S1 and S2). Further sampling from different taxonomic units and additional mitogenomic data will provide a better understanding of the phylogenetic and evolutionary relationships among leafhoppers.

## Conclusions

In the present study, we successfully sequenced the first complete *L. auditura* mitogenome in Ledrinae. To the best of our knowledge, this is the first available mitogenome for a species within the subfamily Ledrinae. The mitogenome is 16,094-bp long, ranging between 15,131 bp of *Ttocnadella arisana* to 16,811 bp of *Parocerus laurifoliae*. Such variations in mitogenome length can be mainly attributed to difference in control region length^25^. Consistent with previous observations in Cicadellidae, the sequences of *L. auditura* mitogenome were highly conserved in terms of gene content, gene size, gene order, base composition, codon usage of PCGs, and RNA secondary structures. Furthermore, there exists a 993-bp-long repeat region between *Cytb* and *ND1*, which contains 2 tandem repeats (Figs 1, 6); the first repeat sequence is 105-bp long and repeated 5 times and the second is 117-bp long and repeated 4 times (Fig. 6). Interestingly, the repeat sequences are located within the control region, similar to that reported in previous studies^9,25,28^. Moreover, we analyzed the mitogenomic features, base composition, codon usage, and phylogenetic relationships of *L. auditura*. In ML and BI analyses, 40 obvious clusters of leafhoppers were identified, consistent with previous phylogenetic findings based on mitogenome data. While Ledrinae was recovered as a paraphyletic group, it emerged as a sister clade to Tartessinae and Iassinae or Aphrodinae, although with low branch support, and its relationship with other clades remained poorly resolved, as revealed by the ML bootstrap analysis of the concatenated anchored hybrid enrichment nucleotide sequence data set in the study of predecessors^35^. There were also large variations in results obtained using different datasets; according to transcriptome analyses, Ledrinae was recovered as a monophyletic group with maximum bootstrap support using ML analyses, with relatively low support among Cicadellidae, and the placements of subfamilies relative to one another were not consistent^38^. Recently, partial mitogenome sequence data were sequenced in leafhoppers, particularly in small groups with few species. Thus, addition of taxa to our small group of mitogenome dataset may help improve the resolution of the still poorly understood relationships among leafhopper lineages. Therefore, the complete mitogenome reported in the present study may provide a basis for further genomic studies of Ledrinae and may be useful for future phylogenetic analyses of Cicadellidae.

## Materials and Methods

### Sample collection and DNA extraction

*L. auditura* specimens were collected from Xianheping, Anlong County, Guizhou Province, China (24°58′N, 105°30′E). Live specimens were preserved in 100% ethanol and stored at −20 °C until DNA extraction. Total genomic DNA was extracted from one adult specimen using the DNeasy© Tissue Kit (Qiagen, Germany). Voucher DNA and specimens (GZU-IHC-000252) are deposited at the Institute of Entomology, Guizhou University.

### Mitogenome sequencing and assembly

*L. auditura mitogenome* was sequenced using next-generation sequencing (Illumina HiSeq. 2500 and 2 GB raw data; Berry Genomics, Beijing, China), and 2 sequence fragments were reconfirmed via polymerase chain reaction (PCR) amplification using primers #2 and #3 (Table 3). We used 40 μL genomic DNA for next-generation sequencing and diluted the remaining genome with ddH_2_O to obtain a concentration of 100 μL for PCR amplification. Primers were designed based on the sequencing results obtained using Primer Premier 6.0 (Premier Biosoft, Palo Alto, CA, USA). PCR was performed using PCR MasterMix (Tiangen Biotech Co., Ltd., Beijing, China) according to the specification manual. The PCG cycling conditions included pre-denaturation at 94 °C for 3 min followed by 30 cycles of denaturation at 94 °C for 30 s, annealing at a suitable temperature for 30 s, elongation at 70 °C for 1 min, and additional elongation at 70 °C for 10 min at the end of all cycles. The annealing temperatures were adjusted according to the melting temperatures of different primers. Table 3 lists primers used in this study. Clean next-generation sequencing results were assembled using Geneious R9^27^ based on the *COX1* fragment (sequenced using primer #1; Table 3) of mitochondrial DNA, and the sequencing results obtained via PCR and TA cloning were assembled using the SeqMan program package (DNAStar Inc.; Madison, WI, USA).

### Sequence analysis and gene annotation

The assembled mitogenome was initially annotated using the MITOS web server with invertebrate genetic codes^39^ and then analyzed using Geneious R9^27^ and NCBI BLAST (https://blast.ncbi.nlm.nih.gov). The locations and secondary structures of 22 tRNAs were identified and predicted using tRNAscan-SE version 1.21^40^ and ARWEN version 1.2^41^. Two rRNA genes were indetified based on the locations of adjacent tRNA genes and then compared with the rRNA genes of other Cicadellidae species. Next, the secondary structures of these rRNAs were predicted based on previously reported models^16,17,25^. DNASIS version 2.5 (Hitachi Engineering, Tokyo, Japan) and RNA Structure version 5.2^30^ were used to predict helical elements present in variable regions. Strand asymmetry was calculated using the following formulas: AT skew = (A − T)/(A + T) and GC skew = (G − C)/(G + C)^42^. Furthermore, base composition and codon usage patterns of PCGs were analyzed using MEGA6^43^. Repeated sequences in the *L. auditura* mitogenome were identified using the Tandem Repeats Finder tool (http://tandem.bu.edu/trf/trf.html)^44^. The complete *L. auditura* mitogenome is deposited in GenBank under the accession number MK387845.

### Sequence alignment and phylogenetic analysis

Phylogenetic analysis was based on 45 Cicadellidae species with 2 Fulgoroidea species (*Ricania speculum* and *Peregrinus maidis*) selected as outgroups (Table S1). Sequences of 13 PCGs (without stop codons) and 2 rRNA genes were used to analyze the phylogenetic relationships. Each PCG and rRNA sequence was aligned using the MAFFT algorithm in Translator X (http://pc16141.mncn.csic.es/index_v4.html)^45,46^ and MAFFT v7.0 online server (https://mafft.cbrc.jp/alignment/server/) using the G-INS-i strategy^47^, respectively. Poorly aligned sequences were eliminated using Gblocks 9.1b (http://www.phylogeny.fr/one_task.cgi?task_type=gblocks)^47^. Finally, all sequences were assessed and manually corrected using MEGA6^43^.

The alignments of individual genes were concatenated to generate 3 datasets including 13 PCGs and 2 rRNAs: (1) amino acid sequences of 13 PCGs (3,366 amino acids); (2) nucleotide sequences of 13 PCGs and 2 rRNAs (11,918 bp); (3) the first and second codons of 13 PCGs and 2 rRNAs (8,552 bp). ML phylogenetic trees were constructed using IQ-TREE v1.6.3^48^, with the best model for each partition selected under the corrected Akaike Information Criteria using PartitionFinder2 (Table S2)^49^, and evaluated using the ultrafast bootstrap approximation approach for 10,000 replicates. Furthermore, BI analysis was conducted using MrBayes 3.2.6^50^; following the partition schemes suggested by PartitionFinder, all model parameters were set as unlinked across partitions. Two simultaneous runs with 4 independent Markov chains were performed for 50 million generations, sampling every 100 generations. After the average standard deviation of split frequencies fell below 0.01, the first 25% samples were discarded as burn-in and the remaining trees were used to generate a consensus tree and calculate the posterior probabilities.

## Supplementary information

Supplementary Information
