# Supplementary material for: Structural and phylogenetic implications of the complete mitochondrial genome of Ledra auditura
Source: Sci Rep. 2019 Oct 31;9:15746. doi: 10.1038/s41598-019-52337-9 (PMC6823449; doi:10.1038/s41598-019-52337-9)
Supplement: Supplementary file 1 — Supplementary Information [file 41598_2019_52337_MOESM1_ESM.pdf]

Structural and phylogenetic implications of the complete mitochondrial genome of *Ledra auditura*

Jia-Jia Wang[1], De-Fang Li[1], Hu Li[1,2], Mao-Fa Yang[1], Ren-Huai Dai[1*]

[1]The Provincial Key Laboratory for Agricultural Pest Management Mountainous Region, Institute of Entomology, Guizhou University; Guiyang, Guizhou 550025 P.R. China

[2]Shaanxi Key Laboratory of Bioresources; Shaanxi University of Technology, Hanzhong, Shaanxi, 723000 P.R. China

***Corresponding author:** Ren-Huai Dai (dmolbio@126.com)

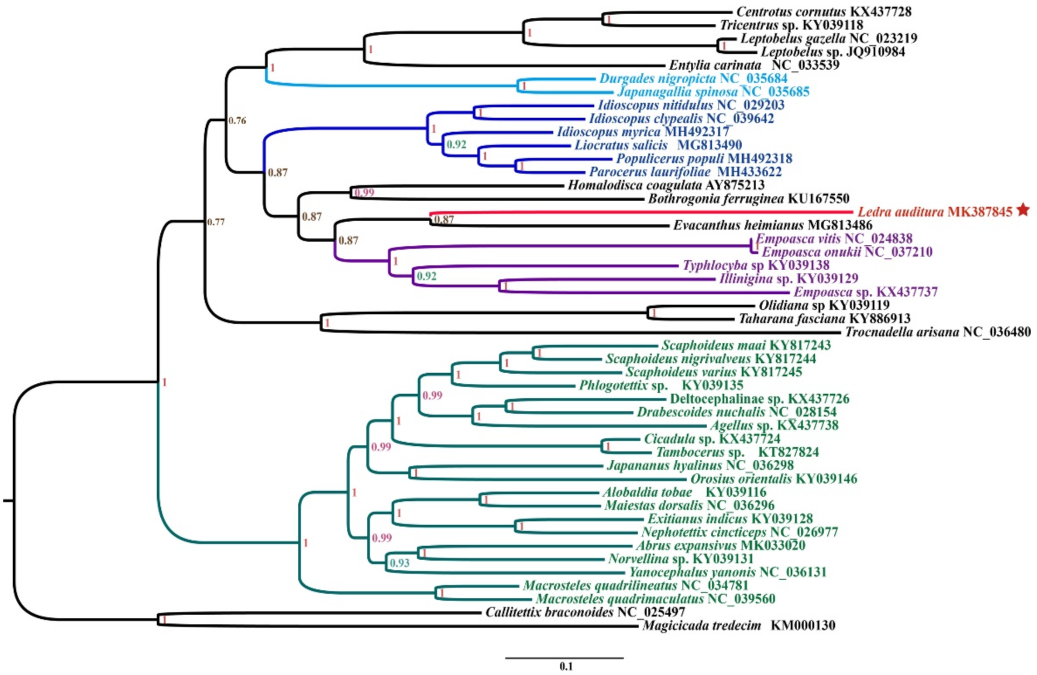

Figure S1 Phylogenetic trees of *Ledra auditura* inferred from amino acids of 13 PCGs using GTR+I+G model in MrBayes.

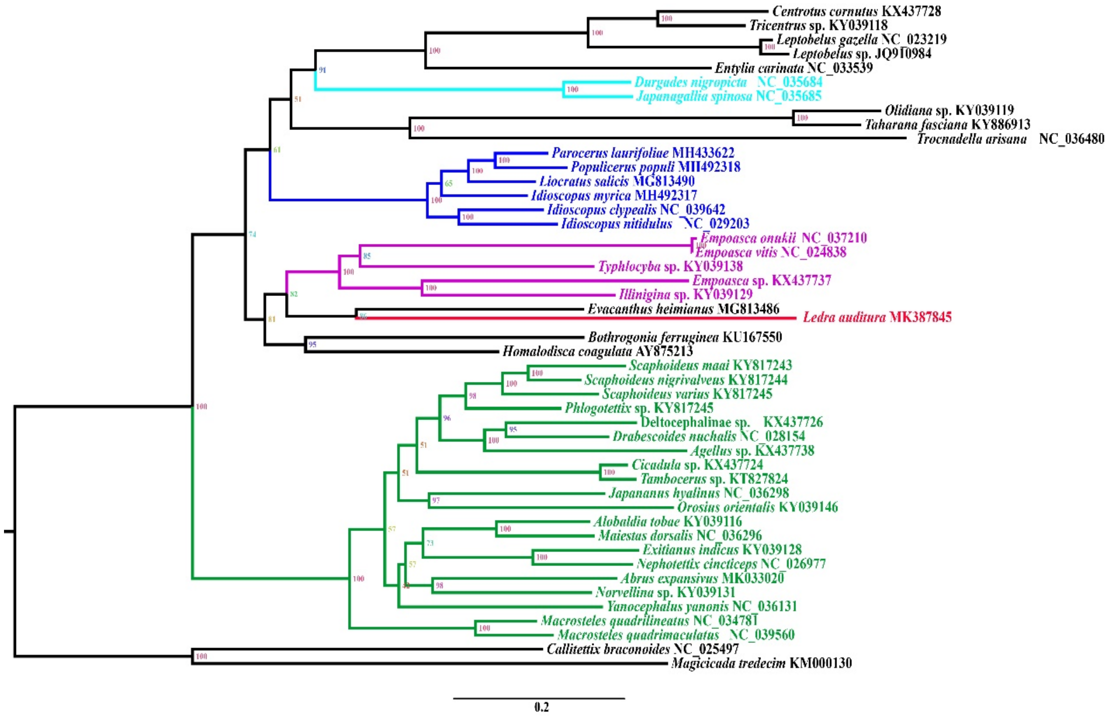

Figure S2 Phylogenetic trees of *Ledra auditura* inferred from amino acids of 13 PCGs using IQ-TREE.

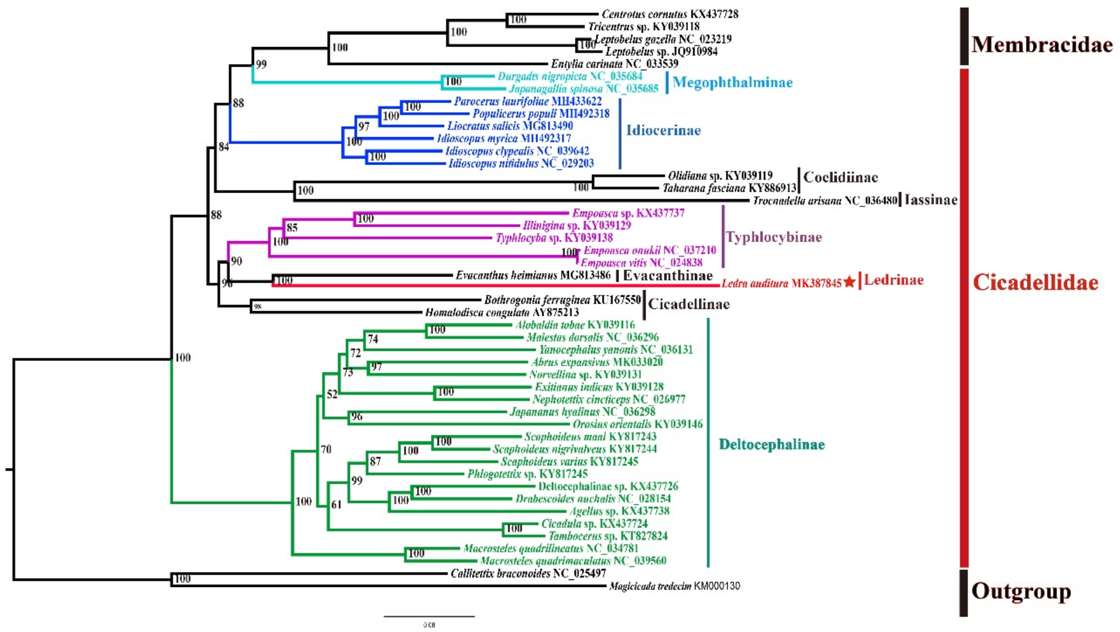

Figure S3 Phylogenetic trees of *Ledra auditura* inferred from the first and second codon positions of the 13 PCGs and 2 rRNAs in IQ-TREE.

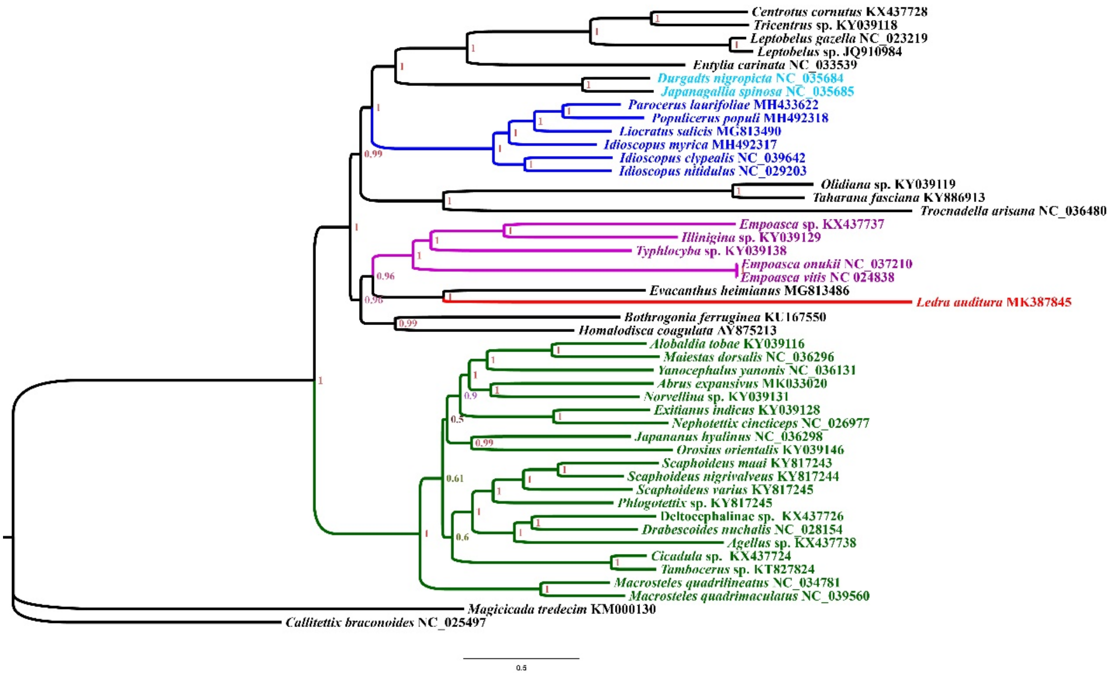

Figure S4 Phylogenetic trees of *Ledra auditura* inferred from the 13 PCGs and 2 rRNAs using GTR+I+G model in MrBayes.

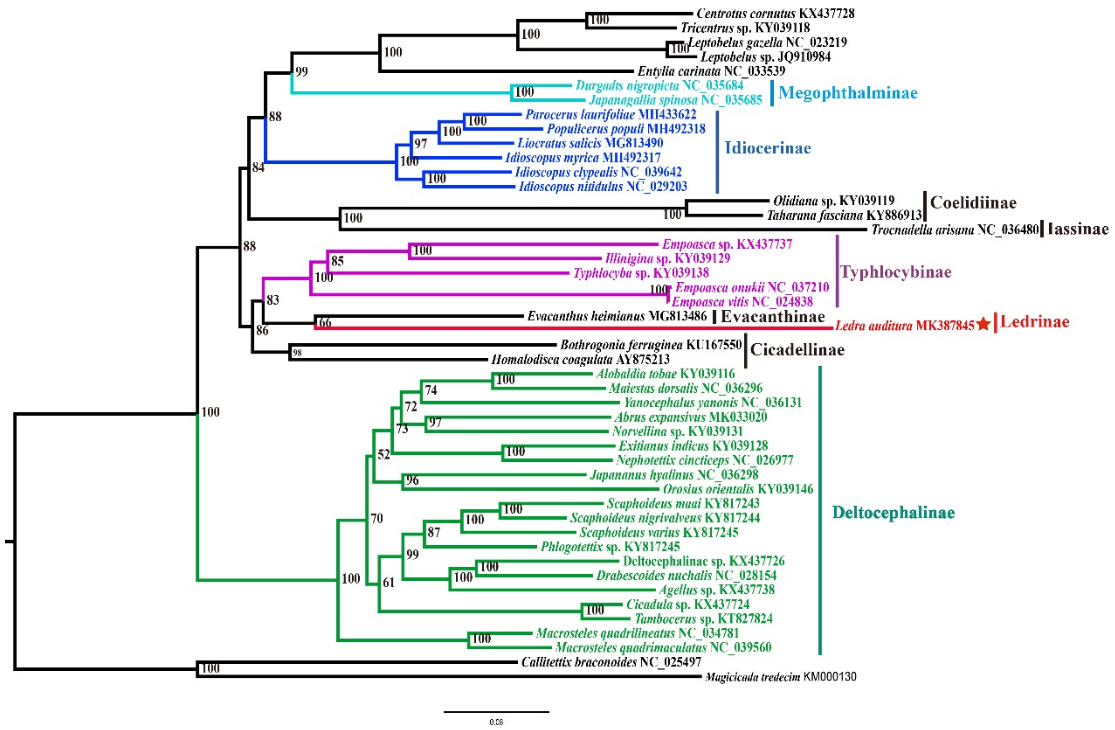

Figure S5 Phylogenetic trees of *Ledra auditura* inferred based on 13 protein-coding genes and 2 rRNA genes in IQ-TREE.

Table S1. Taxonomic information and GenBank accession numbers for the species used in this study.

Family/Subfamily	**Species**	**Accession number**	**Reference**
Deltocephalinae	*Scaphoideus maai*	KY817243	
	Scaphoideus nigrivalveus	KY817244	[1]
	Scaphoideus varius	KY817245	
	Phlogotettix sp 2.	KY039135	[2]
	Deltocephalinae sp.	KX437726	[3]
	Drabescoides nuchalis	NC_028154	[4]
	Agellus sp.	KX437738	[3]
	Cicadula sp.	KX437724	
	Tambocerus sp.	KT827824	[5]
	Alobaldia tobae	KY039116	[2]
	Maiestas dorsalis	NC_036296	[6]
	Yanocephalus yanonis	NC_036131	[2]
	Abrus expansivus	MK033020	Unpublished
	Norvellina sp.	KY039131	[2]
	Exitianus indicus	KY039128	[2]
	Nephotettix cincticeps	NC_026977	Unpublished
	Japananus hyalinus	NC_036298	[6]
	Orosius orientalis	KY039146	[2]
	Macrosteles quadrilineatus	NC_034781	[7]
	Macrosteles quadrimaculatus	NC_039560	[8]
Membracidae	*Centrotus cornutus*	KX437728	[3]
	Tricentrus sp.	KY039118	Unpublished
	Leptobelus gazella	NC_023219	[9]
	Leptobelus sp.	JQ910984	[10]
	Entylia carinata	NC_033539	[11]
Megophthalminae	*Durgades nigropicta*	NC_035684	[12]
	Japanagallia spinosa	NC_035685	
Idiocerinae	*Idioscopus clypealis*	NC_039642	[13]
	Idioscopus nitidulus	NC_029203	[14]
	Idioscopus myrica	MH492317	
	Liocratus salicis	MG813490	[15]
	Parocerus laurifoliae	MH433622	
	Populicerus populi	MH492318	
Typhlocybinae	*Empoasca onukii*	NC_037210	[16]
	Empoasca sp.	KX437737	[3]
	Empoasca vitis	NC_024838	[17]
	Illinigina sp.	KY039129	[2]
	Typhlocyba sp	KY039138	[2]
Evacanthinae	*Evacanthus heimianus*	MG813486	[18]
Ledrinae	*Ledra auditura*	MK387845	This study
Cicadellinae	*Bothrogonia ferruginea*	KU167550	Unpublished
	Homalodisca coagulata	AY875213	
Coelidiinae	*Olidiana* sp	KY039119	Unpublished
	Taharana fasciana	KY886913	[19]
Iassinae	*Trocnadella arisana*	NC_036480	Unpublished
Out group	*Ricania speculum*	NC_031369	[20]
	Peregrinus maidis	NC_037182	[21]

Reference

[1] Y. Du, W. Dai, C.H. Dietrich, Mitochondrial genomic variation and phylogenetic relationships of three groups in the genus *Scaphoideus* (Hemiptera: Cicadellidae: Deltocephalinae). *Sci. rep.* 2017, *7*(1): 16908.

[2] Song N, Cai W, Li H. Deep-level phylogeny of Cicadomorpha inferred from mitochondrial genomes sequenced by NGS. *Sci. rep.* 2017, 7(1): 10429.

[3] N. Song, W.Z. Cai, H. Li, Insufficient power of mitogenomic data in resolving the auchenorrhynchan monophyly. *Zool. J. Linn Soc.* 2017, 183(4): 776-790.

[4] Y.F. Wu, R.H. Dai, H.P. Zhan, L. Qu, Complete mitochondrial genome of Drabescoides nuchalis (Hemiptera: Cicadellidae). *Mitochondrial DNA Part A* 2017, 27(5): 3626-3627.

[5] P.F. Yu, M.X. Wang, L. Cui, X.X. Chen, B.Y. Han, The complete mitochondrial genome of Tambocerus sp. (Hemiptera: Cicadellidae). *Mitochondrial DNA Part A* 2017, 28(1): 133-134.

[6] Y. Du, C. Zhang, C.H. Dietrich, Y. Zhang, W. Dai, Characterization of the complete mitochondrial genomes of Maiestas dorsalis and Japananus hyalinus (Hemiptera: Cicadellidae) and comparison with other Membracoidea. *Sci. Rep.* 2017, 7: 14197.

[7] M. Mao, X.S. Yang, G. Bennett, The complete mitochondrial genome of Macrosteles quadrilineatus (Hemiptera: Cicadellidae). *Mitochondrial DNA Part B* 2017, 2: 173-175.

[8] Y. Du, C.H. Dietrich, W. Dai, Complete mitochondrial genome of Macrosteles quadrimaculatus (Matsumura) (Hemiptera: Cicadellidae: Deltocephalinae) with a shared tRNA rearrangement and its phylogenetic implications. *Int. J. Bio. Macromol.* 2019, 122: 1027-1034.

[9] X. Zhao, A.-P. Liang, Complete DNA sequence of the mitochondrial genome of the treehopper Leptobelus gazella (Membracoidea: Hemiptera). *Mitochondrial DNA Part A* 2016, 27(5): 3318-3319.

[10] H. Li, J.M. Leavengood, E.G. Chapman, D. Burkhardt, F. Song, P. Jiang, J.P. Liu, X.G. Zhou, W.Z. Cai, Mitochondrial phylogenomics of Hemiptera reveals adaptive innovations driving the diversification of true bugs. *P. Roy. Soc. B-Bio. Sci.* 2017, 284(1862): 20171223.

[11] M. Mao, X. Yang, G. Bennett, The complete mitochondrial genome of Entylia carinata (Hemiptera: Membracidae). *Mitochondrial DNA Part B* 2016, 1(1): 662-663.

[12] J.J. Wang, R.H. Dai, H. Li, H.P. Zhan, Characterization of the complete mitochondrial genome of Japanagallia spinosa and Durgades nigropicta (Hemiptera: Cicadellidae: Megophthalminae). *Bioche. Syst. Ecol.* 2017, 74: 33-41.

[13] R.H. Dai, J.J. Wang, M.F. Yang, The complete mitochondrial genome of the leafhopper Idioscopus clypealis (Hemiptera: Cicadellidae: Idiocerinae). *Mitochondrial DNA Part B* 2018, 3(1): 32-33.

[14] J.S. Choudhary, N. Naaz, B. Das, B.P. Bhatt, C.S. Prabhakar, Complete mitochondrial genome of Idioscopus nitidulus (Hemiptera: Cicadellidae). *Mitochondrial DNA Part B* 2018, 3(1): 191-192.

[15] J.J. Wang, M.F. Yang, R.H. Dai, H. Li, X.Y. Wang, Characterization and phylogenetic implications of the complete mitochondrial genome of Idiocerinae (Hemiptera: Cicadellidae). *Int. J. Bio. Macromol.* 2018, 120: 2366-2372.

[16] J.H. Liu, C.Y. Sun, J. Long, J.J. Guo, Complete mitogenome of tea green leafhopper, Empoasca onukii (Hemiptera: Cicadellidae) from Anshun, Guizhou Province in China. *Mitochondrial DNA Part B.* 2017, 2(2): 808-809.

[17] N. Zhou, M. Wang, L. Cui, X.X. Chen, B.Y. Han, Complete mitochondrial genome of Empoasca vitis (Hemiptera: Cicadellidae). *Mitochondrial DNA Part A.* 2016, 27(2): 1052-1053.

[18] J.J. Wang, M.F. Yang, R.H. Dai, H. Li, Complete mitochondrial genome of Evacanthus heimianus

(Hemiptera: Cicadellidae: Evacanthinae) from China. *Mitochondrial DNA Part B.* 2019, 4(1): 284-285.
[19] J.J. Wang, H. Li, R.H. Dai, Complete mitochondrial genome of *Taharana fasciana* (Insecta, Hemiptera: Cicadellidae) and comparison with other Cicadellidae insects. *Genetica.* 2017, 145(6): 593-602.
[20] Q.X. Zhang, D.L. Guan, Y. Niu, L.Q. Sang, X.X. Zhang, S.Q. Xu, Characterization of the complete mitochondrial genome of the Asian planthopper Ricania speculum (Hemiptera: Fulgoroidea: Ricannidae). *Conserv. Genet. Resour.* 2016, 8(4): 463-466.
[21] Y.X. Huang, D.Z. Qin, The complete mitochondrial genome sequence of the corn planthopper, Peregrinus maidis (Hemiptera: Fulgoroidea). *Mitochondrial DNA Part B* 2017, 2(2): 783-784.

Table S2. Partition strategies and evolutionary models used in ML analysis.

Dataset	Subset	Best Model	Site (bp)	Partition names
PCG12RNA 8 partitions	1	GTR+F+R	1062	cox3_pos1, cox2_pos1, cob_pos1, atp6_pos1
	2	TVM+F+R4	1679	cox1_pos2, cox2_pos2, atp6_pos2, nad3_pos2, cox3_pos2, cob_pos2
	3	GTR+F+I+G4	537	cox1_pos1, atp8_pos2, atp8_pos1
	4	GTR+F+I+G4	1282	nad4l_pos1, nad5_pos1, nad4_pos1, nad1_pos1
	5	GTR+F+R4	1282	nad4l_pos2, nad1_pos2, nad4_pos2, nad5_pos2
	6	GTR+F+I+G4	498	nad2_pos1, nad3_pos1, nad6_pos1
	7	TVM+F+G4	392	nad2_pos2, nad6_pos2
	8	GTR+F+R4	1820	rrnL, rrnS
PCGRNA 11 partitions	1	GTR+F+I+G4	712	nad2_pos1, atp8_pos1, atp6_pos1, nad6_pos1, nad3_pos1
	2	TVM+F+I+G4	1692	cox1_pos2, cox3_pos2, atp8_pos2, cob_pos2, cox2_pos2, nad3_pos2, atp6_pos2
	3	TIM3+F+G4	1087	nad3_pos3, nad6_pos3, cob_pos3, atp8_pos3, cox3_pos3, atp6_pos3
	4	GTR+F+I+G4	1372	cox3_pos1, cox2_pos1, cob_pos1, cox1_pos1
	5	TPM3u+F+I+G4	736	cox2_pos3, cox1_pos3
	6	GTR+F+I+G4	1282	nad5_pos1, nad4l_pos1, nad1_pos1, nad4_pos1
	7	GTR+F+I+G4	1282	nad1_pos2, nad4l_pos2, nad5_pos2, nad4_pos2
	8	K3Pu+F+G4	1282	nad4l_pos3, nad4_pos3, nad5_pos3, nad1_pos3
	9	TVM+F+G4	392	nad2_pos2, nad6_pos2
	10	TN+F+ASC+G4	261	nad2_pos3
	11	GTR+F+I+G4	1820	rrnL, rrnS
AA 3 partitions	1	mtART+F+R6	1560	nad2, nad3, nad6, cox3, atp6, cox2, cob
	2	mtART+R5	524	atp8, cox1
	3	mtZOA+F+R6	1282	nad4, nad5, nad1, nad4l
